# Management of a patient with Opalski's syndrome in intensive care unit

**DOI:** 10.1002/ccr3.1111

**Published:** 2017-07-30

**Authors:** Ozer Aynaci, Funda Gok, Alper Yosunkaya

**Affiliations:** ^1^ Department of Anesthesiology and Reanimation Intensive Care Unit Meram Medical Faculty Necmettin Erbakan University 42080 Konya Turkey

**Keywords:** Intensive care unit, Opalski syndrome

## Abstract

Opalski syndrome is a rare vascular brainstem syndrome which is accepted as a variant of Wallenberg syndrome. Opalski syndrome should be considered in acute conditions in which typical symptoms of lateral medullary infarct are accompanied by ipsilateral hemiparesis. Other brain stem syndromes are distinguished from Opalski syndrome by the presence of contralateral hemiparesis.

## Introduction

Wallenberg syndrome is a common clinical picture, which occurs due to the infarct at the lateral part of bulbus and behind inferior olive. Opalski syndrome is a rare variant of Wallenberg syndrome, characterized by hemiparesis presenting with the typical findings of the lateral medullary infarct. This syndrome, which was first described by Opalski in 1946 1, is a very rare brainstem syndrome. Ipsilateral hypoesthesia, Horner syndrome, hemiparesis, hemiataxia, and hypoesthesia findings in the opposite side of the body were described in both conditions 1. Herein, we report a case who was admitted with lateral medullary infarct and ipsilateral hemiparesis, diagnosed with Opalski syndrome, and followed in the intensive care unit.

## Case Presentation

A 75‐year‐old male patient was admitted to the emergency service with fatigue, headache, dizziness, weakness in the left arm and leg, unsteady gait, difficulty in swallowing, dysarthria, and hiccups for 8 h. He had a history of coronary artery disease and hypertension. He was nonsmoker with a long history of high amounts of alcohol consumption.

His blood pressure was found to be 110/60 mmHg in the systemic examination, and his heart was rhythmic. In the transthoracic echocardiogram, ejection fraction was 50%, and no additional pathologies were detected. Laboratory tests showed no other abnormalities except elevated levels of urea and creatinine. Abdominal CT performed in the emergency service showed that left renal artery occlusion and renal atrophy. In his initial neurological examination in the emergency service, he was conscious with speech and comprehension abilities, full cooperation and was able to perform simple tasks. Horner syndrome (ptosis and myositis) and horizontal nystagmus were detected in his left. In the motor examination, proximal and distal areas in the left arm and left leg had a muscle strength of 3/5. He had increased deep tendon reflexes and a positive Babinski reflex on the left. He had contralateral loss of pain and temperature sensation on the left side of his face and right side of his body. Neurological findings suggested that ipsilateral hemiparesis accompanied the typical findings of lateral medullary infarct. Opalski syndrome was considered for the diagnosis, as the clinical presentation was acute, and the patient had the risk factors of stroke.

**Figure 1:**
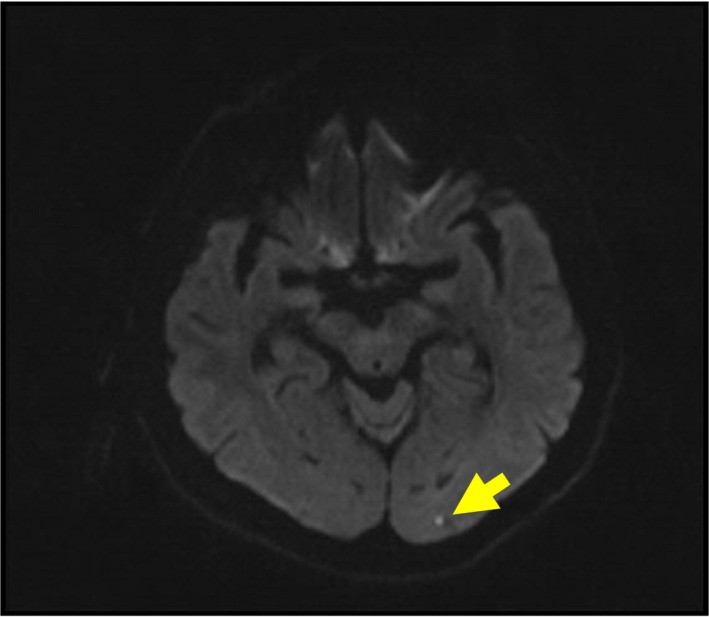
DWI left occipital lacunar infarct.

No pathological findings were detected in computed tomography of the brain. In the diffusion‐weighted image (DWI) of diffusion magnetic resonance imaging (MRI), lacunar lesion concordant with the left occipital hyperintense acute ischemic infarct was observed (Fig. 1). The patient was considered to have ischemic stroke on atherothrombotic base, and antiplatelet treatment (acetylsalicylic acid) was initiated.

**Figure 2:**
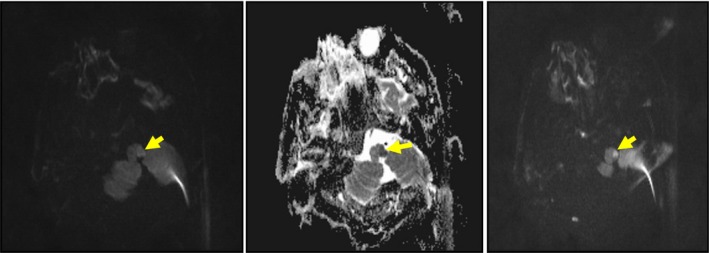
DWI and ADC left bulbar acute ischemic lesion.

The patient had sudden respiratory dysfunction 1 h after his admission to the emergency service and was intubated. When control diffusion MRG was performed, DWI and apparent diffusion coefficient (ADC) map revealed lesion concordant with acute ischemic infarct in the left side of bulbus, in addition to the left occipital lesion (Fig. 2). He was transferred to intensive care unit, and mechanical ventilator treatment was initiated.

In addition, he was administered with low‐molecular‐weight heparin prophylaxis of deep vein thrombosis. The patient was evaluated of delivery of enteral nutrition. There was no contraindication of delivery of enteral nutrition. Enteral nutrition was started through nasogastric tube 24 h after hospitalization. In addition, on the first month of his hospitalization, percutaneous endoscopic gastrostomy was performed, persistent as the swallowing reflex did not recover.

On the fifth day of hospitalization, the patient had new infiltration foci in the radiography, increased secretion, and 38.9°C fever. Tracheal culture was collected using endotracheal intubation. According to the culture results, ventilator‐associated pneumonia diagnosis was made on the 7th day, and antibiotic treatment (piperacillin+tazobactam) was started.

**Figure 3:**
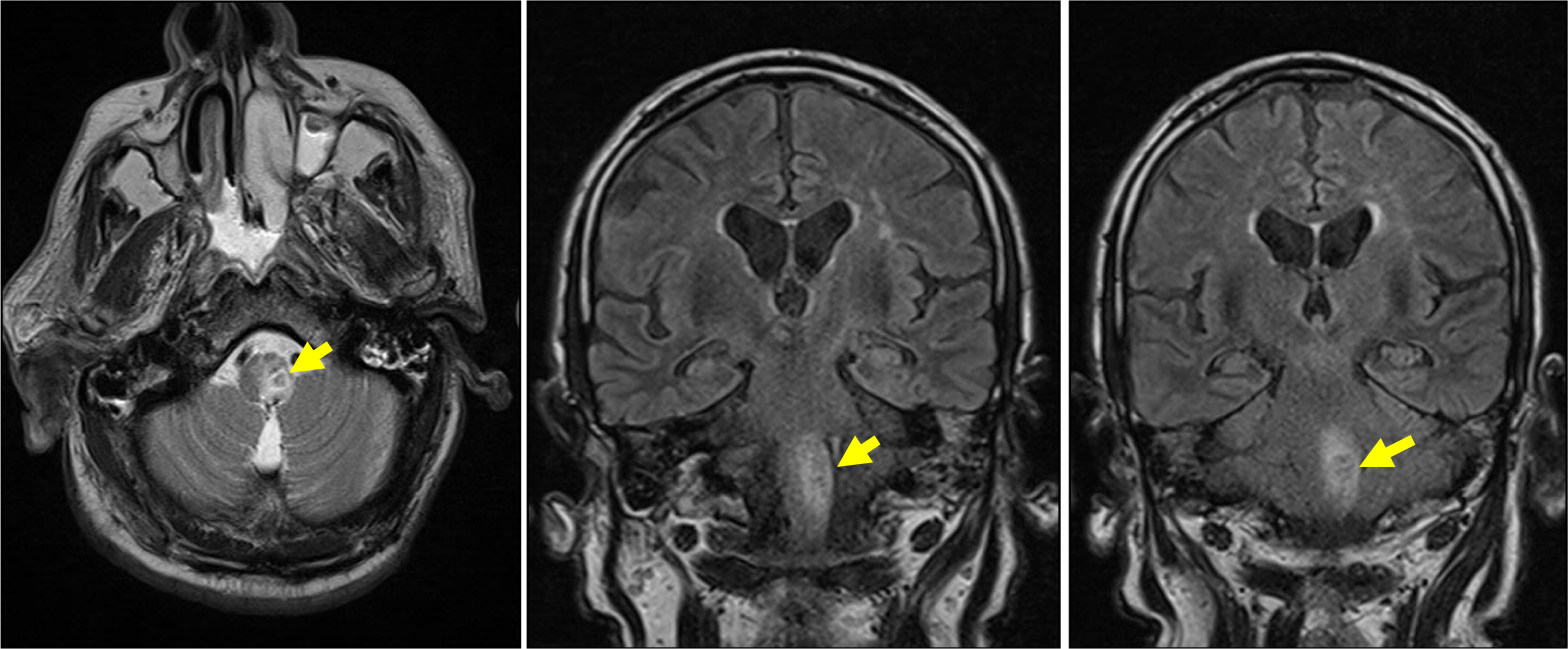
Subacute ischemic lesion in MRI T2 sequence.

**Figure 4:**
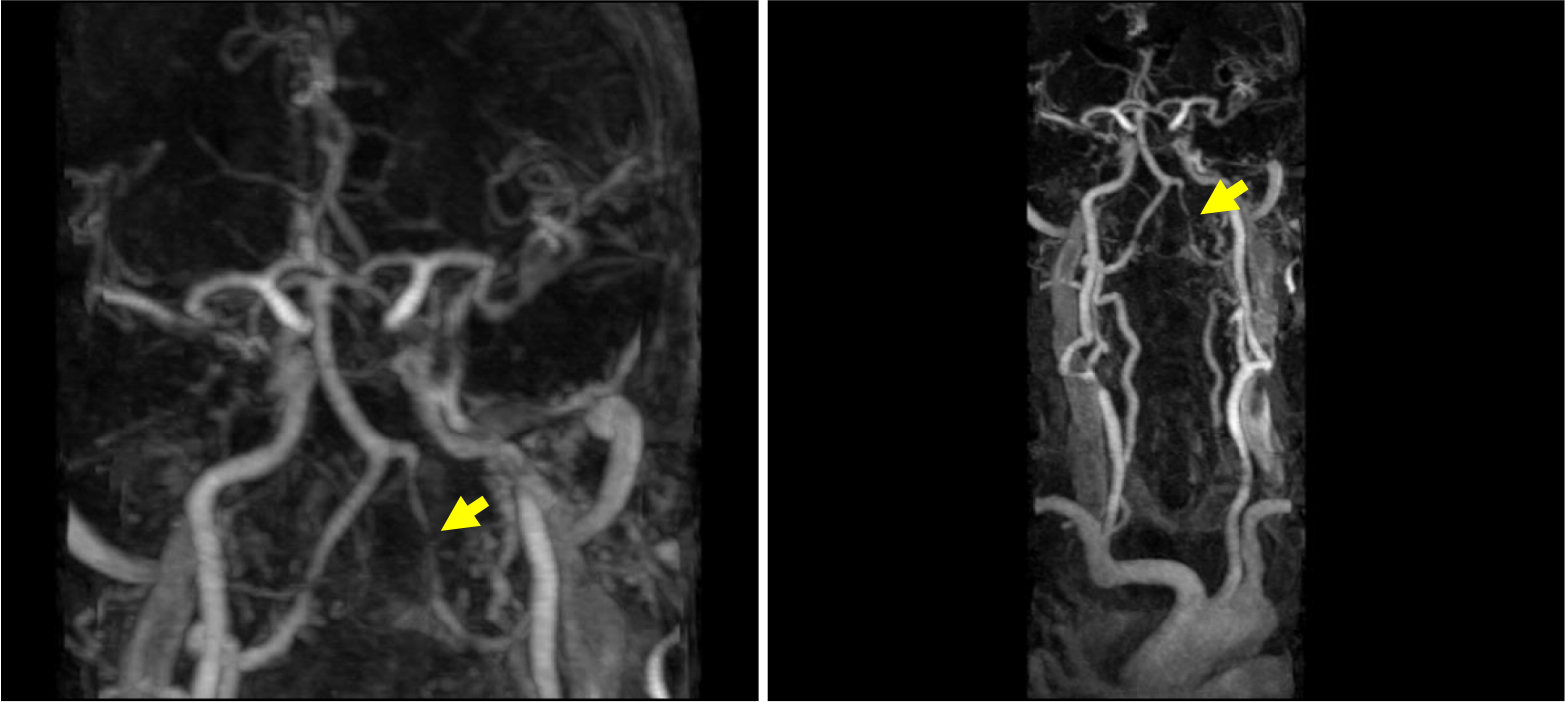
Advanced stenosis in the left vertebral artery visualized in magnetic resonance angiography.

In the motor examination, proximal and distal areas in the left arm and left leg had a muscle strength of 2/5. In the repeated MRI 10 days later, a left bulbar ischemic lesion was detected without any additional lesions (Fig. 3). In the cranial and cervical magnetic resonance angiography (MRA), advanced stenosis was detected in the left vertebral artery (Fig. 4). Antiplatelet treatment was discontinued based on the advice from gastroenterology department, as the patient had melena. In upper gastrointestinal system endoscopy, gastritis was detected on the erythematous base. Two days later, clopidogrel treatment was initiated due to regressed melena.

Patient, who was intubated with mechanical ventilator, was followed up for 11 days. As the weaning cannot be performed during this period, bedside percutaneous tracheostomy was performed. During the follow‐up of the patient in the intensive care unit, the patient was occasionally disconnected from the ventilator for spontaneous breathing trials. Oxygen (4–5 L/min) was delivered only through a T‐piece in tracheostomy. However, during these trials, blood gas collected after a short period (15 min) had a pH of 7.28, partial oxygen pressure (PO_2_) of 53 mmHg, partial carbondioxide pressure (PCO_2_) of 70 mmHg, and HCO_3_ of 26 mEq/L, oxygen saturation (SaO2) 88%. These trials were performed at least four or five times for every other day for a few days. Respiratory depression occurred every time. Thus, it was considered that the patient's need for ventilatory support should be met using mechanical ventilator for a while longer. The patient was provided with a household type mechanical ventilator. Home ventilator settings were adjusted to the same settings with the mechanical ventilator used in the intensive care unit. The patient was discharged to home.

The patient was clinically and hemodynamically stable and was discharged with home mechanical ventilator 2 months after his hospitalization.

## Discussion

The case presented with lateral medullary infarct and ipsilateral hemiparesis and diagnosed with Opalski syndrome was followed in the intensive care unit for a while and was discharged with a home mechanical ventilator.

Opalski syndrome is a rare vascular brainstem syndrome which is accepted as a variant of Wallenberg syndrome. However, the cause and mechanism of hemiparesis in the Opalski syndrome are controversial. Hemiparesis is considered to be caused by the effect of infarct, which extends to caudal region, on corticospinal fibers. Hemiparesis is also considered to be a result of spinocerebellar hypotonic syndrome. Therefore, to diagnose a case with Opalski syndrome, there are views which suggest that pyramidal findings such as positive Babinski reflex should be found on the side with hemiparesis 2. The presence of positive Babinski reflex on the side with hemiplegia in our case is concordant with the Opalski syndrome. In addition to the clinical findings, diagnosis of Opalski syndrome is made upon observing the ischemic lesion extending downwards from the medulla in MRG 3. In our case, the clinical picture was typical. An ischemic lesion extending from medulla to caudal area observed in MRI was consistent with Opalski syndrome (Fig. 3). In definitive diagnosis, Babinski‐Nageotte syndrome with medullary lesion, Marie‐Foix syndrome (Lateral pontine syndrome), and other brain stem syndromes can be considered, but these syndromes are distinguished from Opalski syndrome by the presence of contralateral hemiparesis 4.

Alcohol is among the nonabsolute risk factors for ischemic stroke 5. It was reported that posterior circulation infarcts occur more frequently in those who consume alcohol 6. In our case, the presence of long‐term alcohol consumption history is, therefore, notable.

In our case, MRA examination revealed advanced stenosis in the vertebral artery. The cases reported in the literature are usually occlusion or stenosis of the vertebral artery. However, it was reported that this could be pressure related or a result of dissection 7, 8. In our case, in addition to Opalski syndrome, occipital lacunar infarct was detected. Kim et al. 9 reported a case with cerebellar infarct in addition to Opalski syndrome. It was also reported that lateral medullary infarcts most frequently originate from the vertebral artery.

In cases with Opalski syndrome, the presence of lesion in DWI MRI is rarely reported in the literature. DWI MRG is an invaluable method in the diagnosis of acute ischemic infarct. DWI‐negative findings do not exclude the possibility of brainstem infarct, particularly in the acute stage of medullary lesion. In their study based on patients with lateral medullary infarct, Seo et al. 10 reported that false positivity rate in MR is quite high, particularly within the first 24 h. In addition, there are views which recommend control MRI to document the additional lesions in patients with Opalski syndrome 11. Similar to this case, if the imaging results are not concordant with the clinic, we suggest control MRI to be performed.

In Wallenberg syndrome, automatic respiratory failure due to autonomic dysfunction (Ondine's curse) can be rarely observed 12. This can lead to apnea and death. In this case, respiratory failure after the stroke was considered to be due to the possible autonomic dysfunction. There are not enough data in the literature on respiratory failure in Opalski syndrome. However, sudden deaths due to dystonomy and central hypoventilation can be observed in patients with medullary infarct 13.

In addition, for a patient without GI dysfunction, enteral nutrition should be started within 24 h to prevent further catastrophic consequences 14. Hemodynamically stable patient with normal gastrointestinal system received enteral nutrition through nasogastric tube 24 h after hospitalization. In conclusion, Opalski syndrome should be considered in acute conditions in which typical symptoms of lateral medullary infarct are accompanied by ipsilateral hemiparesis. Other brain stem syndromes are distinguished from Opalski syndrome by the presence of contralateral hemiparesis. While this syndrome is rarely seen, it is a clinical condition that is important to manage during the acute period as it occurs in a vital area such as the brain stem. Therefore, in these cases, we suggest that respiratory failure should be kept in mind, and patients should be followed in the intensive care unit.

## Authorship

OA: prepared manuscript and provided images. FG: helped with literature review and contributed to the manuscript. AY: guided the authors in writing.

## Conflict of Interests

None declared.

## References

[1] Opalski, A. 1946 A new sub‐bulbar syndrome: partial syndrome of the posterior vertebro‐spinal artery. Paris Med. 214:20.

[2] Parathan, K. K. , R. Kannan , P. Chitrambalam , and D. N. Senthil Kumar Aiyappan . 2014 A rare variant of Wallenberg's syndrome: Opalski syndrome. J. Clin. Diagn. Res. 8:MD05. 10.7860/JCDR/2014/9547.4626 PMC4149101 25177595

[3] Bailon, O. , P. Y. Garcia , M. Logak , and S. Timsit . 2011 Opalski syndrome detected on DWI MRI: a rare lateral medullary infarction. Case report and review. Rev. Neurol. 167:177–180. 21087784 10.1016/j.neurol.2010.07.020

[4] Aslanidis, T. , I. Chytas , A. Kontos , and M. Giannakou‐Peftoulidou . 2012 Management of a patient with Opalski's syndrome in intensive care unit and mini review of the literature. Hippokratia 16:373. 23935321 PMC3738616

[5] Djoussé, L. , R. C. Ellison , A. Beiser , A. Scaramucci , R. B. D'agostino , and P. A. Wolf . 2002 Alcohol consumption and risk of ischemic stroke. Stroke 33:907–912. 11935035 10.1161/hs0402.105245

[6] Yang, S. S. , and J. P. Jia . 2013 Differences in risk factors between anterior and posterior circulation affecting young ischemic stroke on set and prognosis. Zhonghuayixuezazhi 93:348–351. 23660206

[7] Dembo, T. , and N. Tanahashi . 2013 Opalski syndrome caused by vertebral artery compression of the lateral surface of the medulla oblongata. Intern. Med. 52:1115–1120. 23676601 10.2169/internalmedicine.52.7177

[8] Deshpande, A. , A. Shetty , A. R. Pai , and S. Rao . 2014 Abnormal brain MRI diffusion‐weighted imaging in a case of Opalski syndrome. BMJ Case Rep.https://doi.org/10.1136/bcr-2013-201695. 10.1136/bcr-2013-201695 PMC3918642 24501334

[9] Kim, H. Y. , S. H. Koh , K. Y. Lee , Y. J. Lee , S. H. Kim , J. Kim , et al. 2006 Opalski's syndrome with cerebellar infarction. J. Clin. Neurol. 2:276–278. 20396533 10.3988/jcn.2006.2.4.276 PMC2854980

[10] Seo, M. J. , S. Y. Roh , Y. S. Kyun , H. J. Yu , and Y. K. Cho . 2006 Diffusion weighted imaging findings in the acute lateral medullary infarction. J. Clin. Neurol. 2:107–112. 20396493 10.3988/jcn.2006.2.2.107 PMC2854949

[11] Kang, K. , L. Jung‐Ju , P. Jong‐Mo , O. Kwon , and K. Byung‐Kun . 2015 Progression of Opalski syndrome to the hemimedullary and contralateral medial medullary infarct. Letter to the editor. Int. J. Stroke 10:E3–E4. 25491550 10.1111/ijs.12385

[12] Pedroso, J. L. , R. F. Baiense , A. P. Scalzaretto , P. B. Neto , A. F. T. de Gois , and M. E. Ferraz . 2009 Ondine's curse after brain stem infarction. Neurol. India 57:206. 19439858 10.4103/0028-3886.51298

[13] Wang, Y. J. , and H. H. Hu . 2013 Sudden death after medullary infarction—A case report. Kaohsiung J. Med. Sci. 29:578–581. 24099113 10.1016/j.kjms.2013.03.002 PMC11916050

[14] Zhang, Z. , Q. Li , L. Jiang , B. Xie , X. Ji , J. Lu , et al. 2016 Effectiveness of enteral feeding protocol on clinical outcomes in critically ill patients: a study protocol for before‐and‐after design. Ann. Transl. Med. 4:308. 27668228 10.21037/atm.2016.07.15 PMC5009025

